# Predictive nomogram for soft robotic hand rehabilitation of patients with intracerebral hemorrhage

**DOI:** 10.1186/s12883-022-02864-2

**Published:** 2022-09-06

**Authors:** Peng Jin, Wei Jiang, Qing Bao, Wenfeng Wei, Wenqing Jiang

**Affiliations:** 1 Department of Neurosurgery, Wujin Hospital Affiliated With Jiangsu University, Changzhou, 213017 Jiangsu China; 2 Department of Neurosurgery, The Wujin Clinical College of Xuzhou Medical University, Changzhou, 213017 Jiangsu China

**Keywords:** Cerebral hemorrhage, Soft robotic hands therapy, S100B, NfL, Nomogram

## Abstract

**Background:**

Few studies focused on the risk factors for hand rehabilitation of intracerebral hemorrhage (ICH) using of soft robotic hand therapy (SRHT). The aim of this study was to establish a predictive nomogram for soft robotic hand rehabilitation in patients with ICH.

**Methods:**

According to the Brunnstrom motor recovery (BMR) stage, the patients were grouped into poor and good motor function groups. The data of patient demographic information and serum level of C-terminal Agrin Fragment (CAF), S100B and neurofilament light (NfL) were collected. The logistic regression was used to analyze the risk factors for poor hand function.

**Results:**

Finally, we enrolled 102 and 103 patients in the control and SRHT groups. For the SRHT group, there were 17 and 86 cases with poor and good motor function at 6-months follow-up respectively. In the good motor function group, the Fugl-Meyer Assessment-Wrist and Hand (FMA-WH score) and BMR score at admission were all better than that in the poor motor function group respectively (*p* < 0.001). The mean serum level of CAF, S100B and NfL in the good motor function group were 2.5 ± 0.82 ng/mL, 286.6 ± 236.4 ng/L and 12.1 ± 10.4 pg/mL respectively, which were lower than that in the poor motor function group (*p* < 0.001, Table 3). The multivariate logistic regression showed that hematoma volume (OR = 1.47, *p* = 0.007), FMA-WH score admission (OR = 0.78, *p* = 0.02), S100B (OR = 1.32, *p* = 0.04), and NfL (OR = 1.24, *p* = 0.003) were all significant predictors of poor motor function.

**Conclusions:**

We found that Soft robotic hands therapy benefited in hand function in patients with ICH and hematoma volume, FMA-WH score admission, S100B, and NfL were all significant predictors for poor motor function of patients with ICH.

## Introduction

The occurrence of intracerebral hemorrhage (ICH) has been steadily growing over the years, and it is still the leading cause of death and long-term impairment globally [1]. Motor skill impairment, including aberrant muscle activation, spasticity, and loss of accuracy, is one of the most impacted areas following ICH [2, 3]. In recent years, an increasing number of neurologists have recognized the necessity of timely intense, task-specific treatment to promote motor recovery for stroke rehabilitation [4–6]. However, the recovery of hand function was complex and difficult because of the non-use in the affected hand. Rehabilitation robots, such as the Hand of Hope and Hand CARE [7], have been widely used in hospitals because of their benefits in assisting physiotherapists therapists in their work. They can provide a series of intensive and repetitive hand function training exercises. However, there is a lack of evidence to show that robot-assisted therapy is more effective than traditional physiotherapy methods, such as Constraint Induced Movement Therapy (CIMT) [8, 9]. Previous studies showed that several factors including neurofilament light (NfL), C-terminal Agrin Fragment (CAF), volume of hematoma, white matter hyperintensities, Alberta Stroke Program Early CT Score (ASPECTS) score, S100B protein would influence the outcome of ischemic stroke rehabilitation [10–14]. While, few studies focused on the risk factors for hand rehabilitation of ICH.

In the study, we examined the role of various factors in restoring hand function after soft robotic hand therapy (SRHT) at 6 months in patients with ICH. The aim of this study was to establish a predictive nomogram for soft robotic hand rehabilitation in patients with ICH.

## Methods

### Patient enrolment

**Fig. 1:**
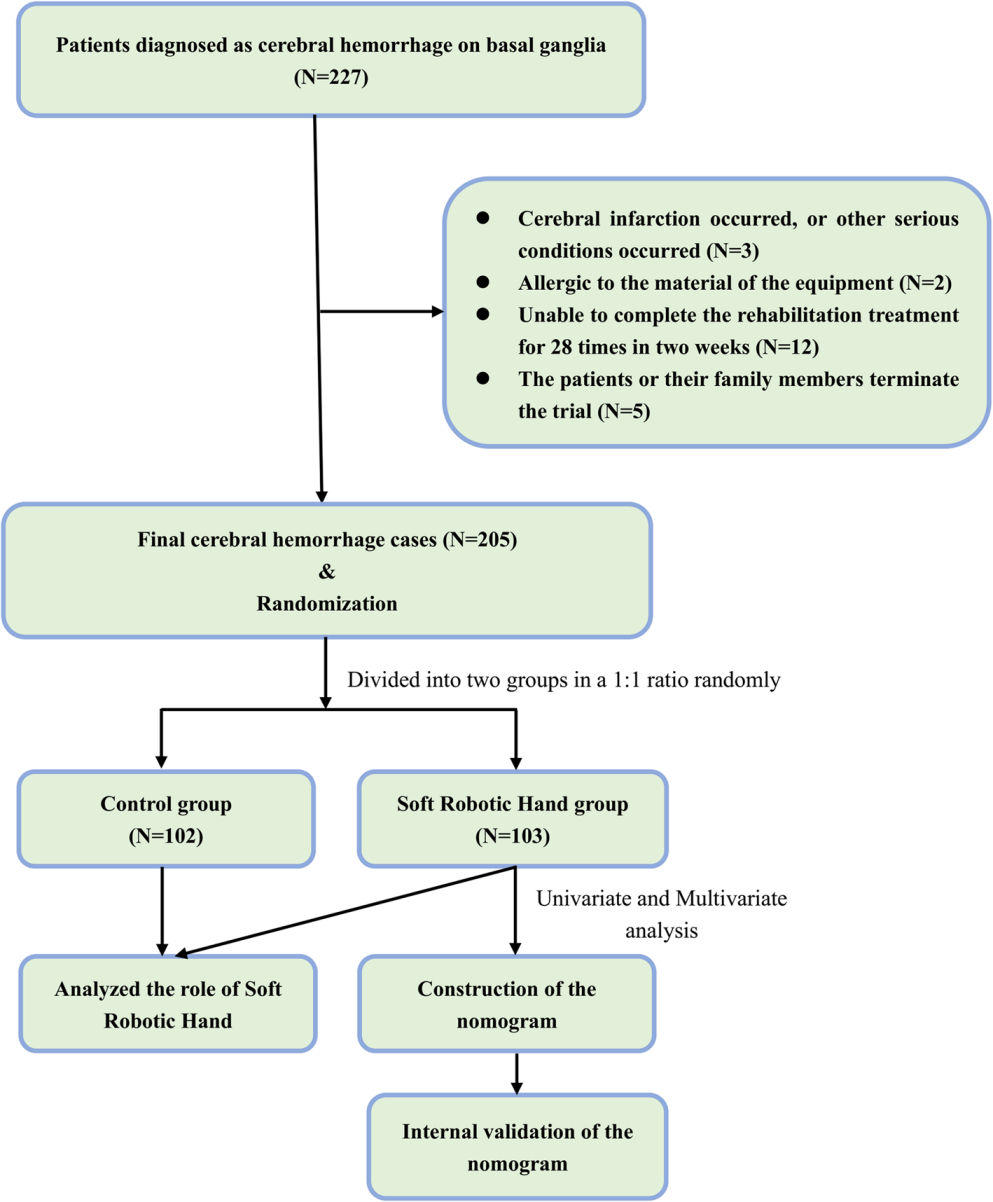
Flow chart for ICH patients with soft robotic hand therapy

The patients with ICH matched the inclusion criteria were enrolled between January 1, 2015 and December 31, 2020 from the Department of Neurosurgery, Wujin Hospital Affiliated with Jiangsu University. All subjects were followed up at least 6 months (Fig. 1).

The inclusion criteria: (1) age range 30 –70 years; (2) No previous history of cerebral hemorrhage and cerebral infarction (3) The lesion was located in the basal ganglia confirmed by CT; (4). bleeding volume range 20 ml -40 ml; (5) any hand dysfunction on wrist stability, finger function, muscle tone and motor function caused by ICH; (6) No severe cognitive dysfunction, depression or anxiety symptoms, the cases can understand and perform the scale test cooperatively; (7) No serious heart, liver, lung or kidney diseases or infectious diseases. (8) The patients and their family are willing to cooperate to complete the rehabilitation treatment for 28 times in two weeks and sign the informed consent.

The exclusion criteria: (1) Cerebral infarction, cerebral hernia, respiratory and circulatory failure occurred; (2) Allergic to the material of the equipment.

### Assessment of hand function

We assessed the hand function of cases before treatment and at the 6-month follow-up. The FMA score and BMR stage were used to evaluate the level of hand motor function.

Patients were divided into good motor function group (BMR stages IV, V, or VI) and poor motor function group (BMR stages I, II, or III) based on their state of motor recovery. In order to ensure the reliability of classifying, two senior physicians assessed the hand function respectively. If they have bifurcation, another senior physician would make the final decision.

### Data collection

Patient demographic information, including age, sex, body mass index (BMI), history of hypertension, diabetes and glasgow coma scale (GCS) at admission were recorded. The locations of the ICH lesions were determined according to the initial CT findings. We used the 3D slicer software to calculate the volume of the ICH. The volume was blindly calculated by two physicians, and these measurements were used to obtain a volume average which was finally used in the analysis.

### Treatment

**Fig. 2:**
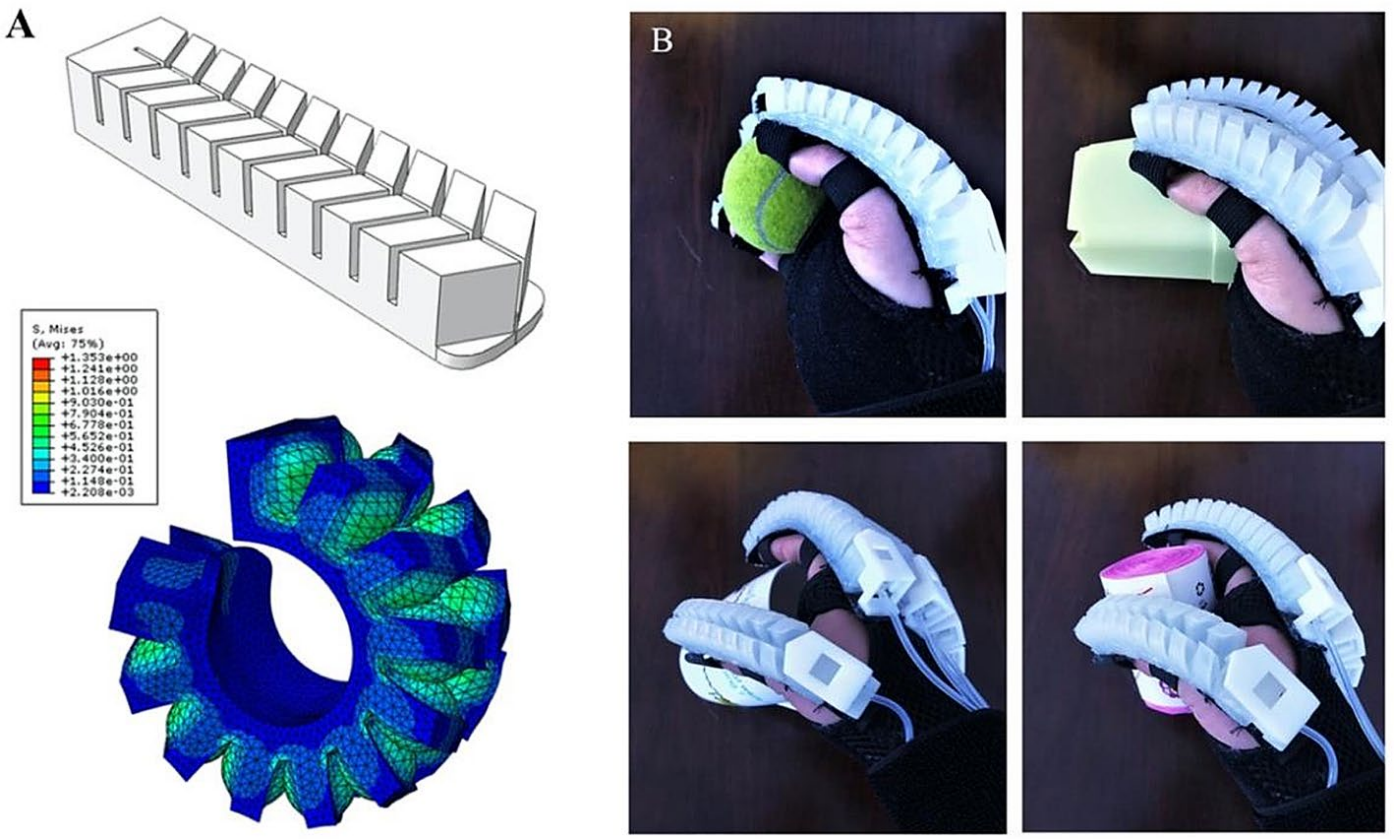
The soft adhesive driver. We adopt soft elastic silicon material to make soft adhesive driver (**A**). After the gas pressure is decompressed, the balloon stretches the finger wearing the device, thus assisting the patient with a complete open and closed hand movement (**B**)

The patients were divided into the SRHT group and the control group according to the therapies. After admission, patients in both groups were treated according to the Guidelines for the Management of Spontaneous Intracerebral Hemorrhage (Stroke. 2015) [15]. The control group got the traditional treatment, which included active, passive range of motion exercises. The SRHT group received the traditional treatment and 40 min of soft robotic hands therapy for each session (5 times each week for 6 weeks). We adopt soft elastic silicon material to make soft adhesive driver (Fig. 2A). An elastic airbag with a special structure is inside the soft rubber driver, and a non-malleable layer to compensate the torque is at the bottom. When air pressure is applied to the air bag, the air bag rolls up towards the side of the strip, causing the driver to bend the finger. After the gas pressure is decompressed, the balloon stretches the finger wearing the device, thus assisting the patient with a complete open and closed hand movement (Fig. 2B).

### Samples and measurement of serum marker

Blood samples obtained at admission were used in analyzing the level of CAF, S100B and NfL respectively. Antecubital venipuncture was used to obtain a 25-mL blood sample. The blood samples were placed in cooled EDTA-treated test tubes (Vacutainers; Becton Dickinson and Company, Sumter, South Carolina) and spun at 2000 g for 15 min. After that, the plasma was decanted into polypropylene tubes containing 500 kallikrein inhibitor units/mL of aprotinin (Sigma-Aldrich Corp, St Louis, Missouri) and refrigerated at -70 °C until analyzed.

The concentrations of CAF, S100B and NfL were analyzed by enzyme-linked immunosorbent assay (ELISA) using commercial kits. Optimal dilutions were determined and ELISAs were performed according to the manufacturer’s instructions. The following kits were used: human CAF ELISA kit (ab216945; Abcam, Cambridge, MA, USA); human S100B ELISA kit (ab234573; Abcam, Cambridge, MA, USA); human NfL ELISA kit (E10709h; EIAab, Wuhan, China).

### Statistical analysis

We reported the categorical and continuous data as percentages and mean standard deviations respectively. We employed the t test or the Mann–Whitney U test for categorical data, and the chi-square test or Fisher's exact tests for continuous variables. We undertook logistic regression procedure to analyze the risk factors for poor motor function. We established a nomogram for predicting poor motor function based on the above risk factors. The plotted decision curves, calibration curves and receiver operating characteristic curve (ROC) were utilized to verify the reliability of nomogram. Statistical significance was set at p-value less than 0.05. The programs R (The R Foundation; http://www.r-project.org; version 3.4.3) was used to analyze the Data.

## Result

### Clinical characteristics summary

Finally, we enrolled 205 patients in this study, 102 in the control group, 103 in the SRHT group. In terms of age, sex, Body Mass Index (BMI), history of hypertension and diabetes, Systolic Blood Pressure (SBP), Diastolic Blood Pressure (DBP), GCS, affected side, hematoma volume and therapy type, the FMA-WH score and BMR score at admission, there were no significant differences between the two groups (*p* > 0.05, Table 1).

**Table 1:** Clinical Summary in the control group and SRHT group

Items	control group (*n* = 102)	SRHT group (*n* = 103)	*P*
**Age(y)**	61.9 ± 12.6	60.3 ± 11.4	0.34
**Sex (Male,%)**	59(57.8%)	69(67.0%)	0.18
**BMI (kg/m**^**2**^**)**	26.0 ± 4.4	26.4 ± 4.5	0.62
**Diabetes (n,%)**	16(15.7%)	13(12.6%)	0.53
**Hypertension (n,%)**	97(95.1%)	99(96.1%)	0.72
**SBP (mmHg)**	167.4 ± 32.2	173.8 ± 31.9	0.16
**DBP (mmHg)**	93.0 ± 18.5	96.2 ± 20.0	0.23
**GCS**	9.8 ± 2.2	10.0 ± 2.0	0.79
**Affected side**			0.73
* Left*	52(51.0%)	55(53.4%)	
* Right*	50(49.0%)	48(46.6%)	
**Hematoma volume (ml)**	26.9 ± 8.6	27.3 ± 8.3	0.79
**Therapy type**			0.37
* Surgery*	51(50.0%)	58(56.3%)	
* Conservative medication*	51(50.0%)	45(43.7%)	

*BMI* Body mass index, *SBP* Systolic blood pressure, *DBP* Diastolic blood pressure, *GCS* Glasgow coma scale, *BMR* Brunnstrom motor recovery, *SRHT* Soft robotic hands therapy, *FMA-WH* Fugl-meyer assessment-wrist and hand

### Functional characteristics before and after rehabilitation

The FMA-WH score at 6-month follow-up in the SRHT group were all significantly higher than that in the control group. The percentage of BMR score VI at 6-month follow-up in the SRHT group were all significantly higher than that in the control group. For both control and SRHT group, the FMA-WH score and BMR score at 6-month follow-up were improved than that at admission (*p* < 0.05, Table 2). For the SRHT group, there were 17 cases with poor motor function and 86 cases with good motor function at 6-months follow up. The mean hematoma volume in the poor motor function group was 34.4 ± 4.2 ml, which was significantly higher than that in the good motor function group (21.2 ± 7.2 ml) (*p* < 0.001). In the good motor function group, the FMA-WH score at admission were all significantly higher than in the poor motor function group, the percentage of BMR score IV/V/VI at admission were significantly higher than that in the poor motor function group (*p* < 0.001).

**Table 2:** The hand function score in the control group and SRHT group

Items	Control group (*n* = 102)	SRHT group (*n* = 103)	*P*
**FMA-WH score at admission**	10.9 ± 6.9	11.4 ± 7.5	0.61
**FMA-WH score at 6-month follow-up**	13.8 ± 7.0	17.4 ± 6.2	< 0.001
*** P***	0.003	< 0.001	
**BMR score at admission**			0.87
* I*	11 (10.8%)	12(11.7%)	
* II*	23(22.5%)	29(28.2%)	
* III*	17(16.7%)	15(14.6%)	
* IV*	21(20.6%)	17(16.5%)	
* V*	12(11.8%)	15(14.6%)	
* VI*	18(17.6%)	15(14.6%)	
**BMR score at 6-month follow-up**			< 0.001
* I*	5(4.9%)	2(1.9%)	
* II*	10(9.8%)	8(7.8%)	
* III*	16(15.7%)	7(6.8%)	
* IV*	17(16.7%)	5(4.9%)	
* V*	21(20.6%)	6(5.8%)	
* VI*	33(32.4%)	75(72.8%)	
***P***	0.01	< 0.001	

*BMR* Brunnstrom motor recovery, *SRHT* Soft robotic hands therapy, *FMA-WH* Fugl-meyer assessment-wrist and hand

### Level of serum marker

The mean serum level of CAF, S100B and NfL in the good motor function group were 2.5 ± 0.82 ng/mL, 286.6 ± 236.4 ng/L and 12.1 ± 10.4 pg/mL respectively, which were lower than that in the poor motor function group (*p* < 0.001, Table 3).

**Table 3:** Clinical Summary in the poor and good motor function group

Items	Good motor function group (*n* = 86)	Poor motor function group (*n* = 17)	*P*
**Age(y)**	60.8 ± 11.0	58.3 ± 12.9	0.41
**Sex (Male,%)**	6(35.3%)	28(32.6%)	0.83
**BMI (kg/m**^**2**^**)**	26.3 ± 4.6	26.7 ± 4.1	0.71
**Diabetes (n,%)**	1(5.9%)	12(14%)	0.36
**Hypertension (n,%)**	17(100%)	82(95.3%)	0.36
**SBP (mmHg)**	172.4 ± 33.2	180.6 ± 23.3	0.33
**DBP (mmHg)**	94.3 ± 19.7	106.2 ± 19.3	0.02
**GCS**	10.0 ± 2.0	9.6 ± 2.2	0.41
**Affected side**			0.96
* Left*	9(52.9%)	46(53.5%)	
* Right*	8(47.1%)	40(46.5%)	
**Hematoma volume (ml)**	21.2 ± 7.2	34.4 ± 4.2	< 0.001
**Therapy type**			0.067
* Surgery*	4(23.5%)	41(47.7%)	
* Conservative medication*	13(76.5%)	45(52.3%)	
**FMA-WH score at admission**	9.2 ± 5.5	4.8 ± 3.5	< 0.001
**BMR score at admission**			< 0.001
* I*	3(3.5%)	9(52.9%)	
* II*	18(20.9%)	7(41.2%)	
* III*	12(14.0%)	0	
* IV*	14(16.3%)	1(5.9%)	
* V*	20(23.3%)	0	
* VI*	19(22.1%)	0	
**CAF (ng/mL)**	2.5 ± 0.82	4.3 ± 0.72	< 0.001
**S100B (ng/L)**	286.6 ± 236.4	571.9 ± 190.8	< 0.001
**NfL (pg/mL)**	12.1 ± 10.4	41.5 ± 6.3	< 0.001

*BMI* Body mass index, *SBP* Systolic blood pressure, *DBP* Diastolic blood pressure, *GCS* Glasgow coma scale, *BMR* Brunnstrom motor recovery, *SRHT* Soft robotic hands therapy, *FMA-WH* Fugl-meyer assessment-wrist and hand

### Nomogram construction and validation

We used the univariate logistic regression to analyze the association between sex, age, BMI, history of diabetes, hypertension, SBP, DBP, GCS, affected side, hematoma volume, therapy type, FMA-WH score admission, BMR score admission, CAF, S100B, NfL and poor motor function. We found that DBP (OR = 1.03, *p* = 0.03), hematoma volume (OR = 1.48, *p* = 0.001), FMA-WH score admission (OR = 0.67, *p* < 0.001), CAF (OR = 8.18, *p* < 0.001), S100B (OR = 1.04, *p* < 0.001), and NfL (OR = 1.17, *p* < 0.001) were all significant predictors of poor motor function (Table 4).

**Table 4:** Univariate and multivariate logistic regression model for predicting poor motor function

Variables	Univariate analysis		Multivariate analysis	
	OR (95% CI)	*P* value	OR (95% CI)	*P* value
**Age(y)**	0.98 (0.94,1.03)	0.41	0.98(0.93,1.03)	0.98
**Sex**				0.55
* Female*	1		1	
* Male*	0.89(0.29,2.64)	0.83	0.55(0.16,1.92)	
**BMI (kg/m2)**	1.02(0.91,1.15)	0.71	1.02(0.89,1.18)	0.68
**Diabetes (n,%)**		0.38		0.30
* No*	1		1	
* Yes*	0.39(0.05,3.18)		3.14(0.36,27.19)	
**Hypertension (n,%)**		0.89		0.52
* No*	1		1	
* Yes*	3.4(0.01,6.78)		1.45(0.53,3.21)	
**SBP (mmHg)**	1.01(0.99,1.03)	0.33	0.98(0.96,1.01)	0.30
**DBP (mmHg)**	1.03(1.00,1.06)	0.03	1.15(0.86,1.23)	0.06
**GCS**	0.89(0.68,1.17)	0.41	0.89(0.66,1.19)	0.45
**Affected side**		0.96		0.64
* Left*	1		1	
* Right*	0.98(0.35,2.78)		1.35(0.43,4.)	
**Hematoma volume (ml)**	1.48(1.22,1.80)	0.001	1.47(1.11,1.94)	0.007
**Therapy type**				
* Conservative medication*	1		1	
* Surgery*	2.96(0.89,9.81)	0.08	3.42(0.95,12.39)	0.06
**FMA-WH score at admission**	0.67(0.56,0.79)	< 0.001	0.78(0.63,0.97)	0.02
**BMR score at admission**				
* I*	1		1	
* II*	4.52(0.02,32.8)	0.98	1.25(0.81,3.04)	0.95
* III*	6.31(0.97,14.1)	0.89	1.03(0.92,1.18)	0.16
* IV*	1.36(0.12,2.35)	0.88	2.94(0.13,5.26)	0.86
* V*	1.12(0.06,3.12)	0.91	1.05(0.38,1.42)	0.18
* VI*	5.12(0.89,11.56)	0.95	3.24(0.62,10.09)	0.56
**CAF (ng/mL)**	8.18(3.37,19.86)	< 0.001	6.21(0.89,23.46)	0.54
**S100B (ng/L)**	1.04(1.02,1.06)	< 0.001	1.32(1.01,3.42)	0.04
**NfL (pg/mL)**	1.17(1.09,1.25)	< 0.001	1.24(1.08,1.44)	0.003

*BMI* Body mass index, *VAS* Visual analogue scale *NDI* Neck disability index, *mJOA* modified japanese orthopedic association, *ROM* Range of motion, *SVA* Sagittal vertical axis

**Fig. 3:**
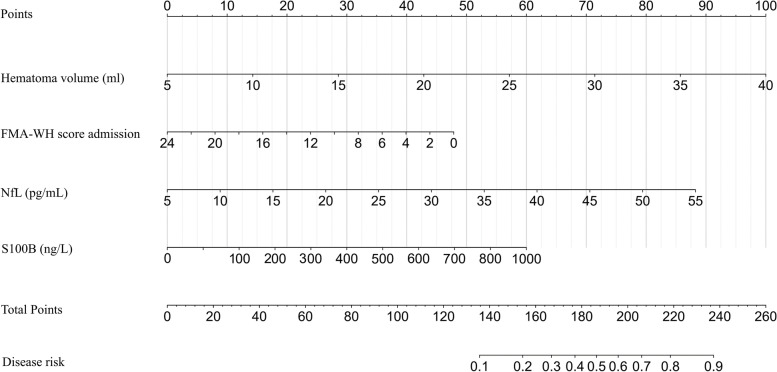
The nomogram to predict poor hand function of ICH patients with soft robotic hand therapy. Based on the risk factors selected, we developed a nomogram to predict poor hand function of ICH patients with soft robotic hand therapy based on the logistic model

Using the multivariate logistic regression, we found that hematoma volume (OR = 1.47, *p* = 0.007), FMA-WH score at admission (OR = 0.78, *p* = 0.02), S100B (OR = 1.32, *p* = 0.04), and NfL (OR = 1.24, *p* = 0.003) were all significant predictors of poor motor function (Table 4). We established the nomogram for predicting poor motor function based on the above risk factors (Fig. 3).

**Fig.4:**
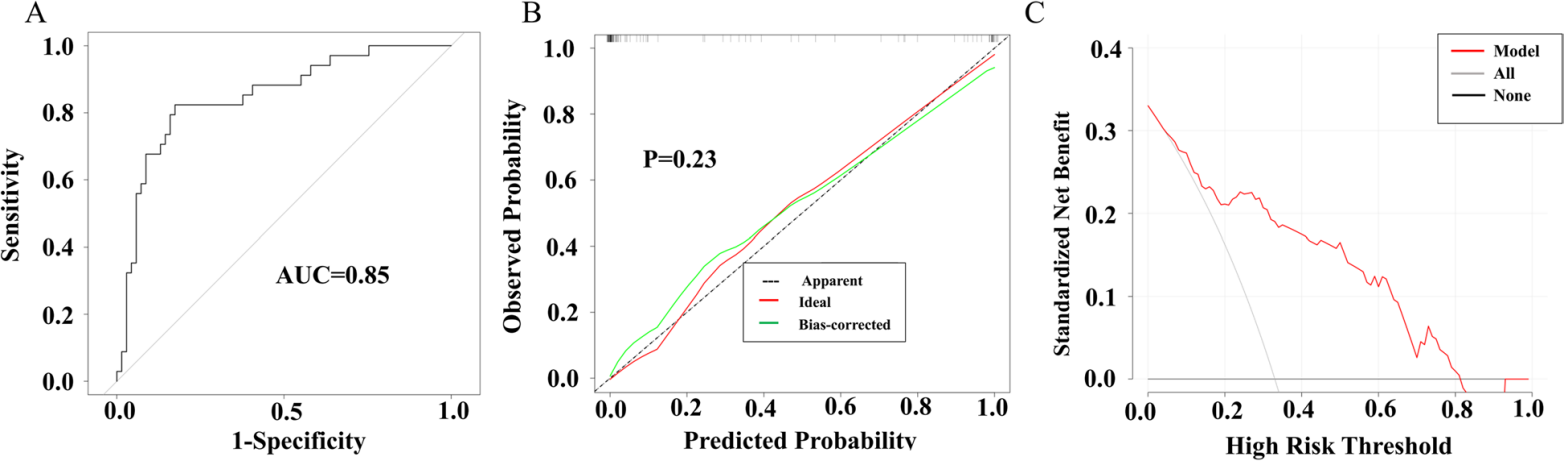
Nomogram Validation. The AUC of the model was 0.85 (**A**), the calibration curves confirmed that the observed outcome fitted nicely to the predicted outcome (*p* = 0.23, **B**). The decision curve showed that if the threshold probabilities were > 10% and < 80%, the nomogram had more advantages than the All or None scheme (**C**)

The area under the curve (AUC) of the model was 0.85 (Fig. 4A), which indicated favorable discrimination. The calibration curves verified that the observed outcome fitted nicely to the predicted outcome (*p* = 0.23, Fig. 4B). The decision curve showed that if the threshold probabilities were > 10% and < 80%, the nomogram had more advantages than the All or None scheme (Fig. 4C). The AUC, accuracy, specificity, sensitivity, positive likelihood ratio (PLR), negative likelihood ratio (NLR), diagnostic odds ratio (DOR) was 0.85, 0.90, 0.86, 0.94, 6.71, 0.07, 96.24 respectively (Table 5).

**Table 5:** Performance of the nomogram in predicting poor motor function

Performance parameter	AUC	Accuracy	Specificity	Sensitivity	PLR	NLR	DOR
Nomogram	0.85	0.90	0.86	0.94	6.71	0.07	96.24

*AUC* Area under the curve, *PLR* Positive likelihood ratio, *NLR* Negative likelihood ratio, *DOR* Diagnostic odds ratio

## Discussion

The first 30 days following a stroke play an important role in beginning recovery [16–18]. However, previous studies showed that only 6% of stroke motor rehabilitation randomized controlled studies recruited all patients during the first month following the stroke [19–21]. The soft robotic hand was with high compliance and low inherent stiffness. Consequently, it is proposed for stroke rehabilitation. While, the clinical efficacy of robot-assisted therapy for stroke rehabilitation was controversial [22–24].

In our study, we undertook logistic regression procedure to analyze the risk factors for poor motor function and established a nomogram. We found that the ICH cases with soft robotic hands therapy had a good hand function at 6 months follow-up. We also found that soft robotic hands therapy benefited in hand function in patients with ICH and hematoma volume, FMA-WH score admission, S100B, and NfL were all significant predictors for poor motor function of patients with ICH.

Previous studies showed that several factors, such as the severity of disability, integrity of the corticospinal tract and the basic state of the patient, would influence the outcome of rehabilitation [25, 26]. Head CT is one of the most commonly used imaging tools to evaluate patients with acute stroke. Branco et al. found that the ASPECTS values ​​showed a strong positive association with upper limb function within the first 24 h of admission, suggesting that the fewer brain regions affected, the more upper limb functions the patient can perform [14]. In our study, the logistic regression found that higher hematoma volume was a significant predictor of poor motor function, which followed the same trend seen in those previous studies. Kelly et al. found that training at home with robot-assisted therapy led to a significant gain on the FMA score, indicating the improved hand function [27]. In our study, we found the similar result, which the logistic regression shown the lower FMA-WH score at admission was a significant predictor of poor motor function.

Several previous studies showed that serum biomarkers such as S100B, NfL, CAF, and Glial fibrillary acidic protein (GFAP) were associated with the prognosis of ischemic stroke, ICH and traumatic brain injury [13, 14, 28]. Branco et al. found that the serum level of S100B was significantly associated with upper limb functioning at 12 weeks [29]. Frankel et al. found that model inclusion of S100B improved prognostic capacity compared with a model containing baseline patient characteristics for the patients’ traumatic brain injury (TBI) [30]. In our study, we found the similar result, which indicated that the higher level of serum S100B was significant predictor of poor motor function. Accordingly, we added the S100B to the prediction model for predicting poor motor function. The S100B is a glial-specific protein expressed predominantly in mature astrocytes. Peng et al. found that the stroke group's serum NfL levels were 9 times higher than those of the healthy control group. He also found that the serum NfL levels were associated with hematoma volume and functional outcome [12]. Pekny found that, in a study on patients with stroke, those who had increased blood levels of NfL had a lower chance of functional improvement [13]. In our study, we also found that the higher level of serum NfL was a significant predictor of poor motor function. Axonal damage results in NfL, an intermediate filament protein, release into the extracellular space. Consequently, the level of NfL in peripheral blood can be used as a biomarker of axon damage and neurodegeneration.

## Conclusion

We found that soft robotic hands therapy benefited in hand function in patients with ICH and hematoma volume, FMA-WH score admission, S100B, and NfL were all significant predictors for poor motor function of patients with ICH. The AUC, calibration curves and decision curve showed that the model was reliable for clinical application.

## Data Availability

The datasets used and/or analysed during the current study available from the corresponding author on reasonable request.
